# How Do Time Since Diagnosis and Sociodemographic Factors Influence Attitudes Towards HIV Status Disclosure in People Living with HIV in Poland? Data from Go Holistic Go Beyond Project

**DOI:** 10.3390/v16111771

**Published:** 2024-11-13

**Authors:** Martyna Lara, Dominik Bursa, Błażej Rozpłochowski, Agata Waszczuk, Monika Bociąga-Jasik, Justyna D. Kowalska

**Affiliations:** 1 Department of Infectious and Tropical Diseases, Jagiellonian University Medical College, 30-688 Cracow, Poland; 2 University Hospital in Cracow, 30-688 Cracow, Poland; 3 Department of Adults’ Infectious Diseases, Medical University of Warsaw, 01-201 Warsaw, Poland; 4 Hospital for Infectious Diseases in Warsaw, 01-201 Warsaw, Poland; 5 Clinic for Acquired Immune Deficiencies, J. Strus Multispecialist City Hospital, 61-285 Poznan, Poland

**Keywords:** HIV status disclosure, stigma, sharing HIV status, HIV testing

## Abstract

The aim of this publication is to present the data from Polish respondents of the Go Holistic Go Beyond Project, which investigates social, professional and intimate relations of people living with HIV in Central and Eastern Europe. We analyze how the patients’ attitude towards disclosing their HIV status changes over time from diagnosis. A questionnaire was distributed among patients by three HIV out-patient clinics. Respondents were compared in three groups defined by the time from diagnosis: over 10 years ago, 6–10 years ago and within 5 years. In total, 381 persons living with HIV participated in the survey, 354 of respondents were male, 23 were female and 4 of the respondents did not identify with any of the above sexes. A significant decrease in hospital-diagnosed cases (from 53% to 39%) was observed, alongside an increased role of private laboratories and voluntary counseling and testing centers. Eighty-nine percent of participants shared their HIV status with at least one social group. There was no significant change in the patterns of HIV status disclosure, reason and form of HIV testing. Our results emphasize the importance of survey-based studies in identifying the needs of people living with HIV in order to improve their general well-being.

## 1. Introduction

Almost twenty thousand people currently live in Poland with HIV infection, 86% of them are on antiretroviral therapy [1]. According to the above-mentioned public payer analyses, in 2010–2022, there was a stable yearly increase of 7% in newly registered patients in HIV care. However, in the year 2022 alone, there have been 2384 newly registered cases of HIV infection in Poland, and this marks a remarkable rise in the number of new diagnoses and can only partly be explained by the incorporation of Ukrainian refugees into national healthcare system [2].

In 2015, UNAIDS defined ambitious targets on the way to end the HIV epidemic by the year 2030, known as ‘95-95-95’. It means that 95% of the people living with HIV (PLWH) know their HIV status, 95% of them receive antiretroviral therapy (ART) and 95% of those on treatment have undetectable HIV viral load [3].

With some countries approaching the UNAIDS targets [4], the fourth pillar of global strategies emerges—the goal to achieve a good Health-Related Quality of Life (HRQoL) in 95% of the virally suppressed PLWH. It represents a holistic approach to perception of health and reflects the perspective of a patient, whose self-perceived well-being is much more multidimensional and goes beyond established laboratory markers like CD4+ cell count and HIV viral load.

Stigma and discrimination on multiple levels is high among people with HIV [5,6]. Prejudice and misconceptions about the virus may be internalized by the person diagnosed with HIV, leading to ‘self-stigma’ and causing adverse medical outcomes (depression, sexual and sleeping problems, etc.) as well as poorer quality of life [7]. Stigmatization around HIV/AIDS topics, rooted in community’s fear of HIV, leads to inadequate prophylactic measures and reluctant attitude towards testing, even in high prevalence populations. It affects linkage to and retention in care as well as adherence to treatment. Therefore, failure to address the ‘fourth 95’ may jeopardize the attainment of the original ‘three 95s’.

Stigma research is gaining attention, and many countries implement various quality of life measurements in routine HIV care [8]. The 40-item HIV stigma scale has been developed [9] and later shortened and adjusted to various populations [10,11]. All of the versions contain a subscale of ‘disclosure concerns’, indicating that considerations about sharing information of HIV infection is a difficult aspect of patient’s adaptation to diagnosis.

According to the ECDC Report, almost 60% of respondents find it difficult to tell people about their HIV infection and 30% of patients feel ashamed of their HIV status [12].

The aim of this publication is to present the data from Polish respondents of the Go Holistic Go Beyond Project, which investigates the social, professional and intimate relations of people living with HIV in Central and Eastern Europe. We analyze how the patients’ attitude towards disclosing their HIV status changes over time from diagnosis.

## 2. Materials and Methods

A panel of experts—physicians specializing in HIV care—created a survey consisting of 7 open-ended and 19 closed-ended questions. The questionnaire was built on SurveyMonkey form and invitations were distributed by three Polish HIV out-patient clinics (in Warsaw, Cracow and Poznan) starting January 2023. Data were exported on 7 November 2023. During this time, all the patients who presented to their clinics were offered enrollment. The study procedure and aims were briefly explained, and the patients were handed a QR code with access to the questionnaire, which they completed at their convenience. Participation was entirely voluntary and anonymous. No exclusion criteria were defined.

Questions in the survey addressed sex, age, place of living, education level, sexual orientation (including heterosexual, homosexual, bisexual and other), relationship status, partner’s HIV status, HIV disclosure history and disclosure recipients (multiple-choice and additional open-ended question), time and place of HIV diagnosis, reasons for testing (multiple-choice and additional open-ended questions to gather extra information), and history of contact with NGOs.

In order to analyze change over time, respondents were compared in three groups defined by self-reported time from diagnosis: over 10 years ago, 6–10 years ago, and within 5 years. Non-parametric (chi-squared) tests were used for group comparisons as appropriate. The statistical analysis was performed using RStudio (version 4.3.1 for Windows).

The study was approved by the Bioethical Committee of Medical University of Warsaw (AKBE/123/2023).

## 3. Results

A total of 381 respondents were included in the study, and 354 (92.91%) of them were male, 23 (6.04%) were female, and 4 of the respondents (1.05%) did not identify with any of the above sexes.

Most of the patients were aged between 30 and 50 years. Unsurprisingly, the older age groups were more numerous in the portion of respondents who were diagnosed with HIV infection earlier.

The majority of our respondents (299 out of 354 men, 84.46%) identified as men who have sex with men (MSM), and the distribution of sexual orientation among the three analyzed groups was even.

The participants were asked about their relationship status. There was a statistically significant predominance of respondents being in longer relationships (>5 years) in the first and second group (diagnosed over 10 years ago and between 6 and 10 years ago).

Our cohort was also analyzed in terms of patients’ education level, their partners’ HIV status and whether they lived, in a big city, a small town or a village—none of the factors yielded statistically significant between-group differences.

The detailed group’s characteristic stratified by the time since diagnosis is depicted in Table 1.

In Table 2, the respondents’ history of HIV testing is scrutinized. Patients diagnosed with HIV earlier usually were tested in a healthcare facility (53.33%, 42.57% and 38.83% in the group diagnosed >10 years ago, 6–10 years ago and <5 years ago, respectively, *p* = 0.04699), while the recently diagnosed patients were more often tested voluntarily in a private laboratory or in a voluntary counseling and testing center (VCT). This tendency is statistically significant. Although there was a trend towards an increased share in HIV diagnosis through other modes of testing, including home-based testing, the proportion was very low.

Various reasons that prompted respondents to get tested for HIV are listed in Table 2, but the answers do not differ significantly in regard to the time since diagnosis. In each group, about half of the participants have undergone the test because they had a history of risky sexual behavior.

Their preferred place of testing was private laboratories and VCTs, similar to the group that did the test because of their symptoms or when starting a new relationship. Conversely, respondents tested by the doctor’s order or due to the positive test in their partner were usually tested in a healthcare facility. The analysis of place of testing stratified by the reason to get tested is presented in Table 3.

In general, 11.08% of respondents admitted that no one knows about their HIV status, and this remains comparable across the three studied groups. Table 4 depicts the tendencies of PLWH toward disclosing the information about their HIV infection grouped by the time since diagnosis. The vast majority of our respondents (84.57–94.44%) in all three groups decided to reveal their HIV status, mostly to their partner and closest family. Our respondents also often shared their HIV status with their friends, and, interestingly, 11.17–13.33% of participants admitted that they do not conceal this information and many people from different groups know about their infection. Two of our patients stated that ‘everyone knows’—one of them was diagnosed within the last 5 years, and the other 6–10 years ago.

Persons with shorter time since diagnosis were less likely to disclose their HIV status (4.44%, 8.91% and 15.43% in the group diagnosed > 10 years ago, 6–10 years ago and in the recent 5 years, respectively, *p* = 0.1847); however, this observation was not statistically significant.

**Figure 1:**
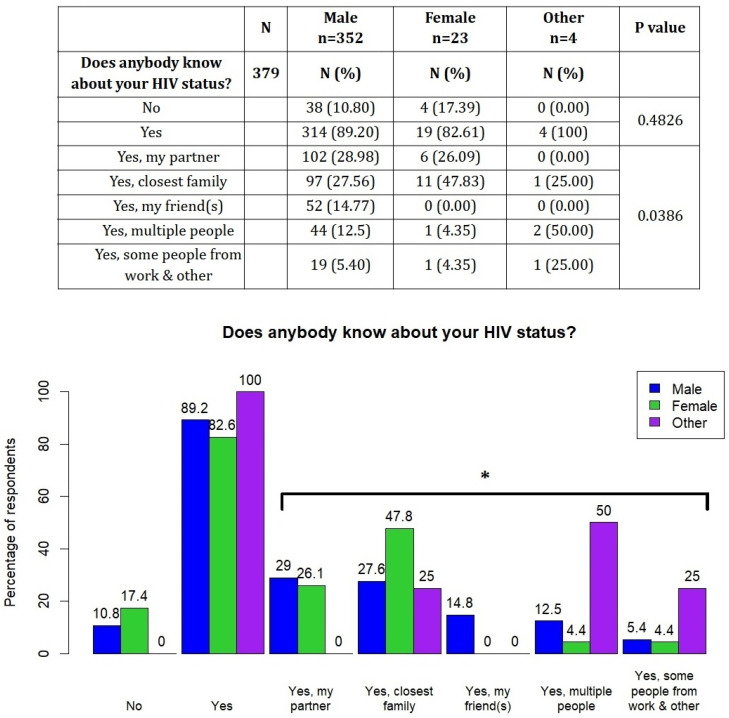
HIV disclosure status stratified by gender. * *p* < 0.05.

We then compared the participants’ responses with regard to their gender in Figure 1. In general, women were slightly more prone than men not to tell anyone about their infection (17.39% of women vs. 10.8% of men, *p* = 0.4826), although there is a substantial difference in the size of both groups (352 men and 23 women). In terms of the specific groups with whom our patients decided to share their HIV status, there were statistically significant differences between the sexes (*p* = 0.0386). Women shared the information with family more often than other groups, while men were the only group to share their HIV status exclusively with friends and also the only one to disclose the information to people from work (depicted collectively with ‘other’).

Men who have sex with men also shared their HIV status more often than heterosexual and bisexual men (90.91%, 72.22% and 80.65%, respectively, *p* = 0.0142).

**Figure 2:**
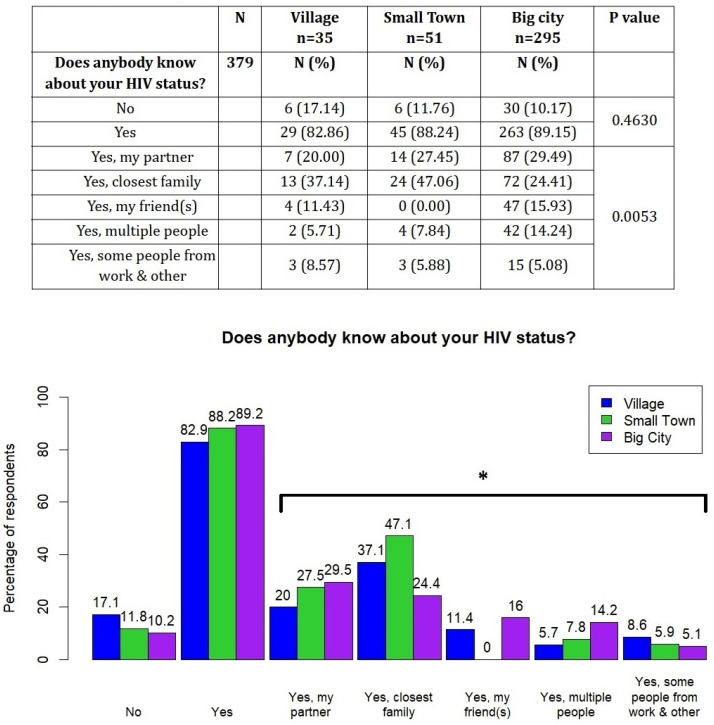
History of revealing HIV status stratified by the place of living. * *p* < 0.05.

Persons living in big cities were more likely to disclose their status, but this observation was not statistically significant (*p* = 0.4630, Figure 2). However, in terms of specific groups, with whom the participants shared their status, there was a significant difference—those who live in small towns or villages more often disclosed their HIV status to family members, while the residents of big cities were more likely to share the information with friends or wider groups of people (*p* = 0.0053, Figure 2).

**Figure 3:**
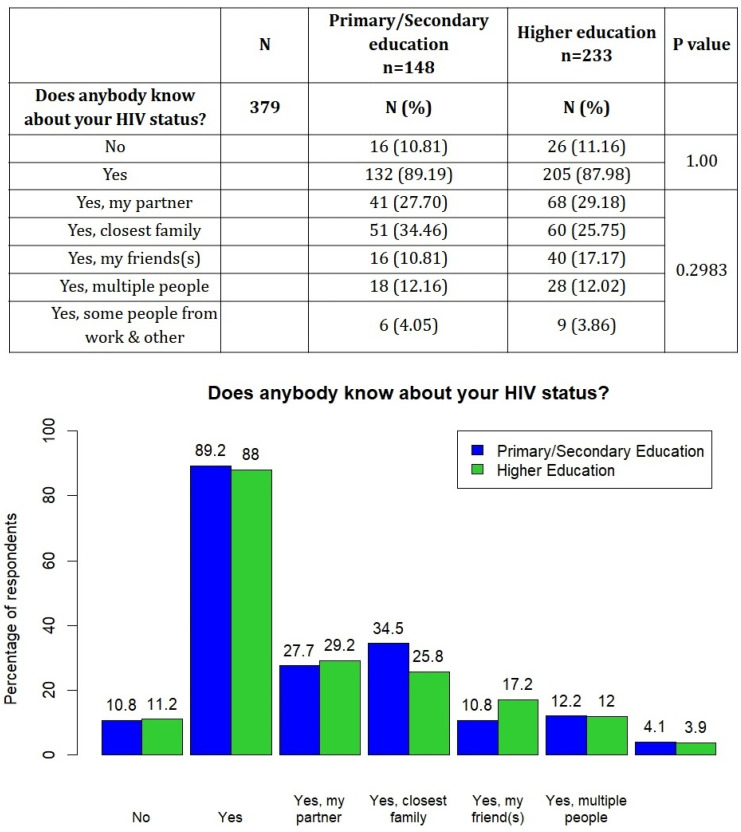
History of revealing HIV status stratified by education level.

The tendency to disclose the HIV status did not depend on the respondents’ educational level (Figure 3).

## 4. Discussion

Almost four hundred patients replied to our survey, which represents around 2% of the Polish population of people living with HIV and being under care in Poland. The three clinics, in which the participants were recruited (in Warsaw, Cracow and Poznan), were the biggest in Poland and together participated in the care of 44% of all Polish HIV patients. The characteristics of people living with HIV who responded to survey did not vary significantly with time since diagnosis, which is in line with analyses presented by NFZ (National Health Fund, public payer), showing that a majority of newly diagnosed patients entering HIV care are young and middle-aged male. Majority of respondents identified themselves as men having sex with men, and the main cause for HIV testing was risky behaviors [1].

The only significant change observed between the three groups stratified by time since diagnosis was age distribution, relationship characteristics, and place of HIV diagnosis. There was no significant change in the patterns of HIV status disclosure, reason and form of HIV test.

Although our findings show similarities with the recent ECDC Stigma Survey [12], medical testing and self-initiated testing at laboratories or VCTs remain the most common mode of diagnosis among people living with HIV and being under medical care in Poland. The contribution of other modes of testing, such as home- or community-based testing, remains insignificant. This is a worrying trend taking into account the high proportion of stigma and discrimination, as well as the shortage of public funds to cover testing and prevention. At the same time, half of the respondents reported to have gotten tested due to risky behaviors, indicating adequate self-awareness of risks and willingness to test.

Self-testing campaigns, in which HIV testing kits are ordered online and delivered directly to consumers, have been organized in Poland exclusively by nonprofit organizations. Demand is consistently high, often exceeding the limited supply. These findings should prompt public health policies to increase free access to home HIV tests, a cost-effective method that requires no healthcare contact and bypasses stigma-related barriers.

Only one in ten persons living with HIV who responded to our survey never disclosed their HIV status to anyone. The recently published data from ECDC stigma survey revealed a higher proportion of people not disclosing their status to anyone [12]. The difference may be explained by a different approach for survey distribution; since in our project we contacted patients in the HIV clinics and in ECDC, distribution was performed through NGOs to a large extent. While patients being under regular follow-up in HIV clinics may have a better acceptance of diagnosis, those seeking help in NGOs may experience self-stigma and be more likely to have negative social experiences in the past.

However, our findings are in accord with the large global meta-analysis, where the disclosure percentage for the European region varied from 85 to 97% [13].

Similar to our results, most commonly the HIV status was disclosed to sexual partners, family and friends. It is also noteworthy that, in Poland, people who are aware of their HIV infection and have a detectable viral load are legally obliged to share this information with their current sexual partners.

In our study, we aimed to define factors that may have influenced respondents’ decision to reveal their HIV status. We found that the time since diagnosis, gender, place of living and education level have not affected the HIV status disclosure rates in general; however, they had, to some extent, shaped the pattern of disclosure recipients.

The time since the diagnosis (>10 years, 6–10 years, and recent 5 years) did not significantly affect the disclosure rates in our respondents. The slight tendency towards sharing the HIV status by those diagnosed earlier cannot be explained solely by the time elapsed since the diagnosis; as according to previous research, most disclosures happen within months up to 3 years from the day of positive HIV test [14,15].

Overall, evidence from the existing literature in that matter is contradictory. Disclosure rates among PLWH vary greatly depending on the time the data were gathered, region of the world, serostatus of the disclosure recipient, and socioeconomic, demographic and cultural factors (like importance of familial relations and criminalization of HIV transmission) [13,14,16].

The fact that HIV infection does not inevitably lead to AIDS and changes observed in the recent years, i.e., the message of U=U (undetectable equals untransmittable), access to pre-exposure prophylaxis and long-acting medications, have changed the landscape for PLWH. Despite the clear benefits of disclosure, these factors certainly eased the burden of responsibility in terms of revealing the HIV status to sexual partners and family.

The only factor that proved statistically significant in relation to overall disclosure rates (disclosure to at least one person vs. no disclosure at all) was sexual orientation. In our cohort, homosexual orientation in men was a facilitating factor, and this result remains consistent when analyzed in men only as well as in the entire group, including other sexes. Analogous observation has been made in some older publications [17,18], but many studies, that assess the impact of sexual orientation on willingness to share serostatus, are confounded by uneven gender representation and inclusion of heterosexual women, are specific to certain ethnic populations or the context of sexual partners only [19]. Higher disclosure rates in this population may be attributed to greater awareness and acceptance of HIV within the gay community and enhanced availability of supportive networks. From this perspective, combined with the fact of increasing rates of heterosexual transmission, it seems reasonable to aim educational campaigns at populations that are not traditionally regarded as ‘key populations’.

According to Positive Perspectives 2 Polish Substudy, none of the Polish patients felt comfortable revealing their HIV status, which is an outstanding observation contrary to those of the overall and European Study subpopulations, where at least few percent of respondents reported feeling comfortable. The main concern of Polish respondents was a fear of being seen or treated differently, fear that someone may disclose their HIV status to others, worries about job loss or how would that information affect their friendships and romantic relationships. Notably, in men, the fear of physical violence was more often reported and in women—concerns about being denied access to healthcare [20]. It needs to be stressed; however, that Polish Perspectives 2 Study included only 50 Polish PLWH, and they differ from our group in terms of the time since diagnosis—few of them were diagnosed in the recent years, while almost a half was diagnosed more than 13 years before the study took place.

Limitations of our work include pre-selection of patients who are already under specialist care and willing to complete the survey. This may underestimate the proportion of people who did not disclose their HIV status. As the survey was distributed via QR code, respondents were not receiving additional explanation to the questions asked. This may result in response bias or misinterpretation. However, our questions were kept short, self-explanatory and used a commonly understandable language. Although our study collected almost four hundred responses, this sample size may not have enough statistical power, especially to analyze separately responses received from women.

## 5. Conclusions

Our results emphasize the importance of survey-based studies identifying social, professional and intimate relations of people living with HIV. Analyses based on such research allow for a better understanding of the quality of life and needs of people living with HIV, as well as tailor interventions resulting in the improvement in general well-being. Our findings on HIV testing should guide health policies to improve awareness of, and access to, self-testing and community-based testing options. Further research is needed to better comprehend factors preventing PLWH from disclosing their HIV status. Addressing these issues in everyday practice can potentially enhance their overall satisfaction with the therapy.

## Figures and Tables

**Table 1:** Group characteristics stratified by the time since diagnosis.

	N	Diagnosed over 10 Years Ago, n = 90	Diagnosed 6–10 Years Ago, n = 101	Diagnosed Within Last 5 Years, n = 188	*p* Value
**Gender**	**381**	**N (%)**	**N (%)**	**N (%)**	
Male		79 (87.78)	99 (98.02)	174 (92.55)	0.06911
Female		10 (11.11)	2 (1.98)	11 (5.85)	
Other		1 (1.11)	0 (0.00)	3 (1.60)	
**Age**	**381**				
20–30		1 (1.11)	9 (8.91) *	60 (31.91)	<0.0001
30–40		26 (28.89)	51 (50.50)	73 (38.83)	
40–50		47 (52.22)	31 (30.69)	45 (23.94)	
50–60		14 (15.56)	9 (8.91)	10 (5.32)	
>60		2 (2.22)	1 (1.00)	0 (0.00)	
* One patient was aged <20 years					
**Sexual orientation**	**381**				
MSM		68 (75.56)	86 (85.15)	143 (76.06)	0.1565
Heterosexual		14 (15.56)	6 (5.94)	20 (10.64)	
Other		8 (8.89)	9 (8.91)	25 (13.30)	
**In a stable relationship**	**381**				
Yes (>5 years)		39 (43.33)	38 (37.62)	50 (26.60)	0.02337
Yes (1–5 years)		13 (14.44)	15 (14.85)	44 (23.40)	
Yes (<1 year)		4 (4.44)	3 (2.97)	17 (9.04)	
No		34 (37.78)	45 (44.55)	77 (40.96)	
**Partners’ status**	**238**				
HIV-positive		22 (24.44)	27 (26.73)	51 (27.13)	0.8656
HIV-negative		36 (40.00)	35 (34.65)	62 (32.98)	
HIV status unknown		1 (1.11)	2 (1.98)	2 (1.06)	
**Where do you live?**	**381**				
Big city		66 (73.33)	83 (82.18)	144 (76.60)	0.1217
Small town		17 (18.89)	13 (12.87)	21 (11.17)	
Village		7 (7.78)	5 (4.95)	23 (12.23)	
**Education**	**381**				
Primary		3 (3.33)	0 (0.00)	7 (3.72)	0.3542
Secondary		32 (35.56)	41 (40.59)	65 (34.57)	
Higher		55 (61.11)	60 (59.41)	116 (61.70)	

**Table 2:** Testing for HIV stratified by the time since diagnosis.

	N	Diagnosed over 10 Years Ago, n = 90	Diagnosed 6–10 Years Ago, n = 101	Diagnosed Within Last 5 Years, n = 188	*p* Value
**Where were you diagnosed?**	**381**	**N (%)**	**N (%)**	**N (%)**	
Healthcare facility		48 (53.33)	43 (42.57)	73 (38.83)	0.04699
Private laboratory		18 (20.00)	16 (15.84)	57 (30.32)	
Voluntary counseling and testing center		17 (18.89)	36 (35.64)	49 (26.06)	
Blood donation		6 (6.67)	5 (4.95)	7 (3.72)	
At home or other		1 (1.11)	1 (0.99)	2 (1.06)	
**Why did you get tested?**	**379**				
I have/used to have risky behaviors		46 (51.11)	47 (46.53)	96 (51.06)	0.8350
Positive result of partner		7 (7.78)	13 (12.87)	25 (13.30)	
Doctor’s order		25 (27.78)	25 (24.75)	44 (23.40)	
New relationship, tested in pregnancy, due to my symptoms, etc.		12 (13.33)	15 (14.85)	23 (12.23)	
**Have you ever been in contact with an NGO?**	**379**				
No		74 (82.22)	75 (74.26)	160 (85.11)	0.8537
Yes		16 (17.78)	25 (24.75)	27 (14.36)	

**Table 3:** Place of HIV testing stratified by the reason to get tested.

	Why Did You Get Tested?					
	N	I Have/Used to Have Risky Behaviors, n = 190	Positive Result of Partner, n = 45	Doctor’s Order, n = 94	New Relationship, Tested in Pregnancy, Due to My Symptoms, etc., n = 50	*p* Value
**Where were you diagnosed?**	**379**	**N (%)**	**N (%)**	**N (%)**	**N (%)**	
Healthcare facility		60 (31.58)	26 (57.78)	68 (72.34)	10 (20.00)	<0.0001
Private laboratory		59 (31.05)	7 (15.56)	10 (10.64)	15 (30.00)	
Voluntary counseling and testing center		66 (34.74)	10 (22.22)	11 (11.70)	15 (30.00)	
Blood donation		3 (1.58)	0 (0.00)	5 (5.32)	9 (18.00)	
Other		2 (1.05)	2 (4.44)	0 (0.00)	1 (4.00)	

**Table 4:** HIV disclosure status stratified by time since diagnosis.

	N	Diagnosed over 10 Years Ago, n = 90	Diagnosed 6–10 Years Ago, n = 101	Diagnosed Within Last 5 Years, n = 188	*p* Value
**Does anybody know about your HIV status?**	**379**	**N (%)**	**N (%)**	**N (%)**	
No		4 (4.44)	9 (8.91)	29 (15.43)	0.1847
Yes		85 (94.44)	92 (91.09)	159 (84.57)	
Yes, my partner		28 (31.11)	31 (30.69)	49 (26.06)	0.8217
Yes, closest family		32 (35.56)	28 (27.72)	49 (26.06)	
Yes, my friend(s)		9 (10.00)	13 (12.87)	30 (15.96)	
Yes, multiple people		12 (13.33)	13 (12.87)	21 (11.17)	
Yes, some people from work and other		4 (4.44)	7 (6.93)	10 (5.32)	

## Data Availability

Data are contained within the article.
